# Habituation of the responsiveness of mesolimbic and mesocortical dopamine transmission to taste stimuli

**DOI:** 10.3389/fnint.2014.00021

**Published:** 2014-03-04

**Authors:** Maria A. De Luca

**Affiliations:** ^1^Department of Biomedical Sciences, Neuropsychopharmacology Section, University of CagliariCagliari, Italy; ^2^National Institute of Neuroscience, University of CagliariCagliari, Italy

**Keywords:** habituation, dopamine, nucleus accumbens, medial prefrontal cortex, taste stimuli, microdialysis

## Abstract

The presentation of novel, remarkable, and unpredictable tastes increases dopamine (DA) transmission in different DA terminal areas such as the nucleus accumbens (NAc) shell and core and the medial prefrontal cortex (mPFC), as estimated by *in vivo* microdialysis studies in rats. This effect undergoes adaptive regulation, as there is a decrease in DA responsiveness after a single pre-exposure to the same taste. This phenomenon termed habituation has been described as peculiar to NAc shell but not to NAc core and mPFC DA transmission. On this basis, it has been proposed that mPFC DA codes for generic motivational stimulus value and, together with the NAc core DA, is more consistent with a role in the expression of motivation. Conversely, NAc shell DA is specifically activated by unfamiliar or novel taste stimuli and rewards, and might serve to associate the sensory properties of the rewarding stimulus with its biological effect (Bassareo etal., 2002; Di Chiara etal., 2004). Notably, habituation of the DA response to intraoral sweet or bitter tastes is not associated with a reduction in hedonic or aversive taste reactions, thus indicating that habituation is unrelated to satiety-induced hedonic devaluation and that it is not influenced by DA alteration or depletion. This mini-review describes specific circumstances of disruption of the habituation of NAc shell DA responsiveness (De Luca etal., 2011; Bimpisidis etal., 2013). In particular, we observed an abolishment of NAc shell DA habituation to chocolate (sweet taste) by morphine sensitization and mPFC 6-hydroxy-dopamine hydrochloride (6-OHDA) lesion. Moreover, morphine sensitization was associated with the appearance of the habituation in the mPFC, and with an increased and delayed response of NAc core DA to taste in naive rats, but not in pre-exposed animals. The results here described shed light on the mechanism of the habituation phenomenon of mesolimbic and mesocortical DA transmission, and its putative role as a marker of cortical dysfunction in specific conditions such as addiction.

## INTRODUCTION

Primary motivational states, both positive and negative, are often ruled by the activity of dopamine (DA) neurons in the ventral tegmental area (VTA) and their terminal targets, such as the nucleus accumbens (NAc) and the medial prefrontal cortex (mPFC). In these terminal regions, DA responds to appetitive or aversive stimuli differently depending on specific factors such as stimulus valence, stimulus sensory modality, specific DA neuron subpopulations, different terminal areas studied, and the techniques used for the detection of DA (e.g., microdialysis vs voltammetry; Fibiger and Phillips, 1988; Di Chiara, 1995; Westerink, 1995; Berridge and Robinson, 1998; Schultz, 1998; Redgrave et al., 1999; Di Chiara et al., 2004; Aragona et al., 2009; Lammel et al., 2012; McCutcheon et al., 2012).

The direct correlation between motivational stimulus valence and its effect on the responsiveness of DA transmission has been extensively appreciated by *in vivo* brain microdialysis studies in three different DA terminal areas: NAc shell, NAc core, and mPFC (Bassareo and Di Chiara, 1999; Bassareo et al., 2002). Particularly, it has been observed that the exposure to natural rewards (e.g., highly palatable food) and to salient food taste stimuli (sweet and bitter) increases DA transmission in NAc shell and core and in mPFC of non-food-deprived rats. In NAc shell, but not in NAc core or in mPFC, this response undergoes adaptive regulation after a single pre-exposure to the same taste/food. This response reduces following a recurrent stimulus, and is termed habituation (Thompson and Spencer, 1966; Cohen et al., 1997; Rankin et al., 2009). In NAc shell, habituation to natural rewards is taste specific, and it is reversed by food deprivation of the animals and modified by the presentation of cues associated with the stimulus (Bassareo and Di Chiara, 1999). These observations demonstrate that NAc shell DA is activated by unfamiliar appetitive taste stimuli while DA in the mPFC codes for generic motivational value independently of stimulus valence. Additionally, this underlines the role of NAc shell DA and its habituation in associative learning (Bassareo et al., 2002; Di Chiara et al., 2004).

In contrast, habituation of DA response is not present after repeated exposure to drugs of abuse (e.g., nicotine, opiates, psychostimulants, cannabinoids), which preferentially stimulate DA transmission in NAc shell as compared to NAc core (Pontieri et al., 1995,^1996^; Tanda et al., 1997). However, the use of *in vivo* voltammetry by other labs showed opposite and specific sub-regional changes in DA concentration in response to both cued and unconditioned appetitive stimuli or after cocaine (Aragona et al., 2009; Brown et al., 2011; Badrinarayan et al., 2012).

This review describes experimental evidence for the disruption of habituation of NAc shell DA responsiveness to motivational stimuli *in vivo,* and on the specific circumstances that could contribute to these significant changes. The data here discussed highlight the role of DA in both learning and hedonic processes.

## SENSITIZATION TO MORPHINE AFFECTS HABITUATION OF MESOLIMBIC AND MESOCORTICAL DOPAMINE RESPONSIVENESS TO TASTE STIMULI

Morphine administration increases DA transmission in the mesolimbic system, as estimated by *in vivo* brain microdialysis (Di Chiara and Imperato, 1988; Pontieri et al., 1996). Specific experimental protocols of repeated exposure to morphine produced sensitization.

The effect of morphine sensitization on the habituation of the responsiveness of DA transmission to a single pre-exposure to novel, remarkable and unpredictable taste stimuli has been evaluated (De Luca et al., 2011). In order to induce behavioral and biochemical sensitization, a protocol conceived by Cadoni and Di Chiara (1999) has been used. Thus, rats were administered twice a day for three consecutive days with increasing doses of morphine (10, 20, 40 mg/kg s.c.) or saline. After 15 days of withdrawal, rats were administered a precise amount of appetitive sweet chocolate solution through an intraoral cannula (1 ml/5 min, i.o.) during the microdialysis session for NAc shell, core and mPFC dialysate DA analysis.

**FIGURE 1:**
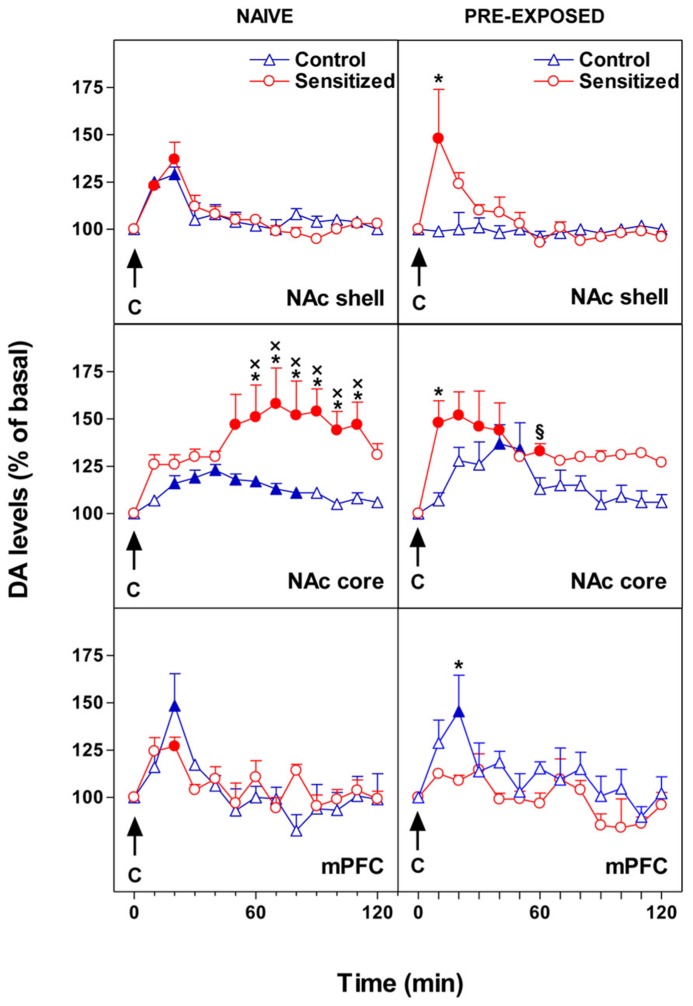
**Effect of 24-h pre-exposure to chocolate (C, 1 ml/5 min, i.o.) on NAc shell and core and mPFC dialysate DA in morphine sensitized or control rats.** Results are indicated as mean ± SEM of change in DA extracellular levels expressed as the percentage of basal values. Solid symbol, *p* < 0.05 vs basal value; *, *p* < 0.05 vs Control; *x*, *p* < 0.05 vs shell naive Sensitized; § , *p* < 0.05 vs shell pre-exposed Sensitized. (Adapted from Figures 2 and 3; De Luca et al., 2011).

Our main finding was that opiate sensitization and chocolate pre-exposure exert a differential influence on the response of DA transmission as regards to the specific subdivision of the mesocorticolimbic DA system. **Figure 1** shows the effect of morphine sensitization on the response of NAc shell and core and mPFC DA levels to intraoral sweet chocolate in naive and chocolate pre-exposed rats. We reported that pre-exposure to chocolate produced opposite changes in DA transmission in the mPFC and in the NAc shell (De Luca et al., 2011). In fact, unexpected appearance of habituation in mPFC DA responsiveness to taste stimuli was accompanied by a loss of habituation in NAc shell. Moreover, morphine sensitization was associated with an increased and delayed (50–110 min after chocolate) response of NAc core DA to taste in naive rats while an immediate increase of DA was observed in pre-exposed animals. Similar results were obtained with an aversive stimulus (De Luca et al., 2011). Moreover, although sensitization to morphine is associated with long-term changes in mesolimbic and mesocortical DA responsiveness to taste stimuli, changes in behavioral taste reactivity are lacking. The latter evidence supports the hypothesis that taste-hedonia does not depend on DA (Berridge and Robinson, 1998), thus the increase of DA transmission in these brain regions could arise from the motivational and not from the sensory or hedonic properties of the taste (Bassareo and Di Chiara, 1999; Bassareo et al., 2002).

All of the DA terminal regions studied displayed changes in the habituation (i.e., abolishment vs appearance), which might result in an increased incentive arousal and learning. Notably, the habituation of mPFC DA responsiveness to chocolate releases NAc shell DA from inhibition, thereby abolishing the single-trial habituation of DA. Under this condition, repeated approaches toward a motivational stimulus might be facilitated.

## THE ABLATION OF THE MPFC DOPAMINE TERMINALS AFFECTS HABITUATION OF MESOLIMBIC DOPAMINE RESPONSIVENESS TO TASTE STIMULI

In intact brain, mPFC DA prominently regulates the activity of subcortical DA areas involved in reward and motivation through a complex interaction of many different sub-regions inside the PFC (Murase et al., 1993; Taber and Fibiger, 1995; Kennerley and Walton, 2011). Such control is modulated by DA receptors in the mPFC (Louilot et al., 1989; Jaskiw et al., 1991; Vezina et al., 1991; Lacroix et al., 2000). mPFC DA functions are engaged in cognitive processes (Seamans and Yang, 2004), regulation of emotions (Sullivan, 2004), working memory (Khan and Muly, 2011), and executive functions such as motor planning, inhibitory response control and sustained attention (Fibiger and Phillips, 1988; Granon et al., 2000; Robbins, 2002).

**FIGURE 2:**
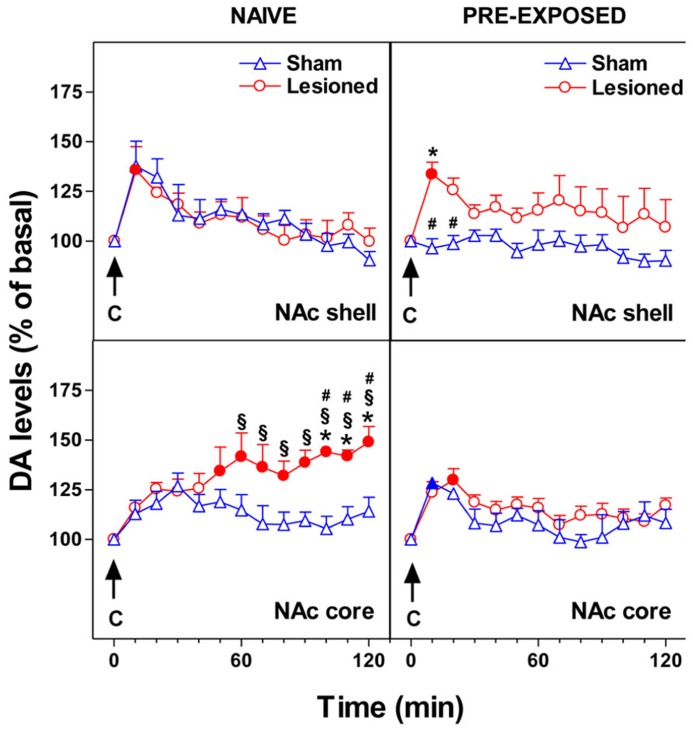
**Effect of 24-h pre-exposure to chocolate (C, 1 ml/5 min, i.o.) on NAc shell and core dialysate DA in 6-OHDA lesioned in the mPFC or control rats.** Results are indicated as mean ± SEM of change in DA extracellular levels expressed as the percentage of basal values. Solid symbol, *p* < 0.05 vs basal value; *, *p* < 0.05 vs Sham; #, *p* < 0.05 vs Lesioned pre-exposed; § , *p* < 0.05 vs Sham pre-exposed. (Adapted from Figure 6; Bimpisidis et al., 2013).

We recently studied the effect of mPFC 6-OHDA lesion on NAc shell and core DA responsiveness to chocolate in naive and chocolate pre-exposed rats. 6-OHDA bilateral infusions in the mPFC modify the responsiveness of NAc DA to gustatory stimuli administered by an intraoral catheter. As shown in **Figure 2**, we observed that in NAc shell of naive subjects the lesion did not change the DA response to intraoral chocolate. However, the lesion of mPFC DA terminals produced an elevated, delayed, and prolonged increase of DA in NAc core in response to an appetitive taste stimulus. In pre-exposed subjects, the lesion did not affect NAc core DA responsiveness to chocolate while it abolished one-trial habituation of NAc shell DA response to sweet taste. After DA terminal lesions, an effect on neither hedonic taste score nor motor activity has been observed (Bimpisidis et al., 2013).

These observations might suggest that the mPFC DA inhibitory control of DA responsiveness in subcortical striatal areas is different depending on the ventral striatum sub-region studied. Moreover, different sub-regions within the mPFC (e.g., prelimbic, infralimbic) have different projections to different compartments of the NAc. Accordingly, in the NAc shell, which is mostly innervated by the infralimbic area, the cortical-subcortical relationship might work in an opposite manner to that in NAc core.

This is consistent with the different responsiveness of NAc shell and core DA to discrete stimuli and conditions (Di Chiara et al., 2004; Di Chiara and Bassareo, 2007; Aragona et al., 2009; Corbit and Balleine, 2011; Cacciapaglia et al., 2012).

## CONCLUSION

The experimental results here described may help explain, in part, the reason why traumatic PFC injury often facilitates development of drug use disorders (Delmonico et al., 1998). Accordingly, disruption of PFC functions appears following both traumatic conditions (Bechara and Van Der Linden, 2005) and history of drug addiction (Van den Oever et al., 2010; Goldstein and Volkow, 2011). Our data also suggest a correlation between the NAc DA responsiveness to repeated exposure to a motivational stimulus and the control of its activity by the mPFC DA. This refers to mPFC a crucial role in subcortical dysfunction, which may occur in different stages of drug addiction. Similarly, the mPFC plays a crucial role in subcortical dysfunction, which may occur in different stages of drug addiction. Other studies show the direct involvement of mPFC in addiction (Schenk et al., 1991; Weissenborn et al., 1997; Bolla et al., 2003), drug seeking, craving and relapse, which are related to drugs taken either by humans or animals (Kalivas and Volkow, 2005).

Remarkably, we found similarities between the effect of repeated morphine exposure and selective mPFC DA terminal lesions on DA transmission in response to motivational taste stimuli both in NAc shell and in NAc core. However, this correlation seems to exist only after prolonged administration of drugs of abuse, as a single drug exposure did not modify the habituation in NAc shell (De Luca et al., 2012). Moreover, the absence of any relationship between DA habituation and taste reactivity (Berridge, 2000; Bassareo et al., 2002; De Luca et al., 2012) has been validated.

In summary, the specific conditions leading to the abolishment of habituation illustrated in this work clarify the meaning of the habituation phenomenon of mesolimbic and mesocortical DA transmission. Habituation is usually present in NAc shell, but not in NAc core or mPFC, and it is ruled by intact DA transmission within the mPFC. However, the appearance of habituation in the mPFC could be considered as a marker of mPFC dysfunction in its ability to inhibit crucial subcortical functions. This may result in excessive motivation for inappropriate actions originating from a clear loss of impulse control. Finally, yet importantly, NAc DA habituation may be considered *per se* as a marker of drug dependence and its liability.

## Conflict of Interest Statement

The author declares that the research was conducted in the absence of any commercial or financial relationships that could be construed as a potential conflict of interest.

## Abbreviations

C: chocolate
DA: dopamine
i.o.: intraorally
mPFC: medial prefrontal cortex
NAc: nucleus accumbens
6-OHDA: 6-hydroxy-dopamine hydrochloride
s.c.: subcutaneously
VTA: ventral tegmental area
